# Case report: Electrocardiographic changes in pembrolizumab-induced fatal myocarditis

**DOI:** 10.3389/fimmu.2023.1078838

**Published:** 2023-02-16

**Authors:** Kazuhiro Nishiyama, Kei Morikawa, Yusuke Shinozaki, Junko Ueno, Satoshi Tanaka, Hajime Tsuruoka, Shinya Azagami, Atsuko Ishida, Nobuyuki Yanagisawa, Yoshihiro J. Akashi, Masamichi Mineshita

**Affiliations:** ^1^ Division of Respiratory Medicine, Department of Internal Medicine, St Marianna University School of Medicine, Kawasaki, Kanagawa, Japan; ^2^ Department of Pathology, St. Marianna University School of Medicine, Kawasaki, Kanagawa, Japan; ^3^ Division of Cardiology, Department of Internal Medicine, St. Marianna University School of Medicine, Kawasaki, Kanagawa, Japan

**Keywords:** irAE, lung cancer, myocarditis, pembrolizumab, COVID-19

## Abstract

Immune checkpoint inhibitor (ICI)-induced myocarditis is rare but fatal. Because of the rapid course of ICI-induced myocarditis, understanding of clinical course is only possible through information from case reports. We report a case of pembrolizumab-induced myocarditis in which we were able to document the course of electrocardiographic changes from onset to death. A 58-year-old woman with stage IV lung adenocarcinoma, who had completed her first cycle of pembrolizumab, carboplatin, and pemetrexed, was admitted with pericardial effusion. She underwent pericardiocentesis after admission. A second cycle of chemotherapy was administered 3 weeks after the first cycle. Twenty-two days after admission, she developed a mild sore throat and tested positive for SARS-CoV-2 antigen. She was diagnosed with mild coronavirus disease 2019 (COVID-19), isolated, and treated with sotrovimab. Thirty-two days after admission, an electrocardiogram showed monomorphic ventricular tachycardia (VT). Suspecting myocarditis caused by pembrolizumab, the patient was started on daily methylprednisolone after coronary angiography and endocardial biopsy. Eight days after the start of methylprednisolone administration, she was considered to have passed the acute stage. However, four days later, R-on-T phenomenon triggered polymorphic VT and she died. The impact of viral infections such as COVID-19 on patients be treated with immune checkpoint inhibitors is still unknown and we need to be careful with systemic management after viral infections.

## Introduction

The advent of immune checkpoint inhibitors (ICIs) has revolutionized cancer treatment. ICIs sustain T-cell activation and exert the anti-tumor effects by blocking immunosuppressive signaling from antigen-presenting cells and tumor cells (1). Currently, seven ICIs are approved for the treatment of cancer. Specifically, they are pembrolizumab, nivolumab (PD-1 inhibitors), atezolizumab, durvalumab, avelumab (PD-L1 inhibitors), ipilimumab, and tremelimumab (CTLA-4 inhibitors). ICIs have shown efficacy in the treatment of lung cancer, but they also cause various immune-related adverse events (irAEs). Among them, myocarditis is rare but has the highest mortality rate among all irAEs (2). In cancer therapy, the incidence of myocarditis has been reported to be 1.14% for all ICIs, 0.5% for PD-1 inhibitors, 2.4% for PD-L1 inhibitors, and 3.3% for CTLA-4 inhibitors (3). In the KEYNOTE-189 trial, which evaluated the efficacy and safety of platinum doublet and pembrolizumab combination chemotherapy in patients with non-squamous non-small cell lung cancer, myocarditis was reported in only one case (0.2%) (4).

Recently, the coronavirus disease 2019 (COVID-19) pandemic has had a major impact on healthcare. COVID-19 is an acute respiratory illness caused by infection with severe acute respiratory syndrome coronavirus 2 (SARS-CoV-2). It is known that some COVID-19 patients develop cytokine release syndrome (CRS), in which inflammation-inducing cytokines are increased and the immune system is activated (5-7). Theoretically, COVID-19 infection could further activate the immune system of cancer patients being treated with ICI, resulting in severe irAEs. We report a case of pembrolizumab-induced myocarditis that developed after COVID-19 infection, in which we were able to document the course of electrocardiographic changes from onset to death.

## Case presentation

**Figure 1:**
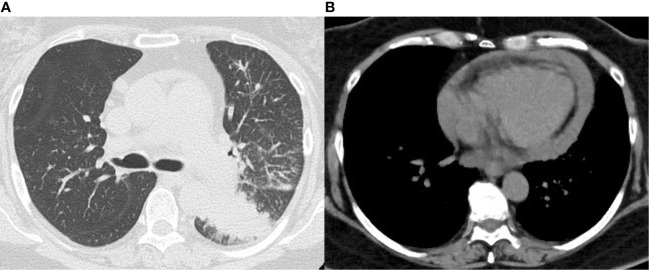
Chest computed tomography on admission. **(A)** Lung window shows a mass shadow in the lower lobe of the left lung. **(B)** Mediastinal window shows pericardial effusion.

A 58-year-old female with a smoking history of at least 35 pack years had no medical history of dyslipidemia, diabetes mellitus, hypertension or other medical conditions, and no family history of coronary artery disease. She received her second COVID-19 vaccination 6 manths ago and no other vaccinations. She was diagnosed with left lower lobular adenocarcinoma of the lung that had metastasized to the left hilar and right mediastinal lymph nodes, invading the pericardium. The tumor was negative for epidermal growth factor receptor (EGFR), anaplastic lymphoma kinase (ALK), with a programmed death ligand 1 (PD-L1) tumor proportion score (TPS) of 25%. She visited her previous physician complaining of dyspnea after completing her first cycle of pembrolizumab, carboplatin, and pemetrexed 9 days earlier. Subsequently, she was referred to our hospital due to a worsening pericardial effusion on computed tomography (CT) scan (**Figure 1**).

**Figure 2:**
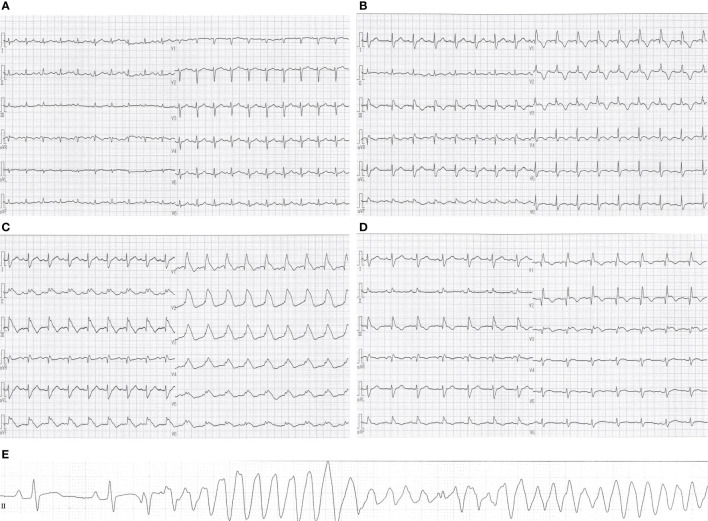
Changes in electrocardiographic waveforms during hospitalization. The admission electrocardiogram showed **(A)** sinus tachycardia and low-voltage QRS complexes. **(B)** Negative T waves appeared on electrocardiogram 27 days after admission, and **(C)** monomorphic VT appeared 5 days later. Eight days after the start of methylprednisolone administration, **(D)** negative T waves remained but ST-segment elevation was no longer present. Pre-death electrocardiogram showed **(E)** R-on-T phenomenon triggered polymorphic VT. QT/QTc intervals: **(A)** 313/443 ms, **(B)** 395/507 ms, **(C)** 433/572 ms, **(D)** 408/482 ms, **(E)** 400/408 ms.

On initial examination, she was afebrile with a blood pressure of 125/78 mm Hg, a heart rate of 124 beats/min, and an oxygen saturation of 97% on 3 liters per minute of oxygen administration. Blood tests showed no elevation of creatine kinase (CK), creatine kinase–myocardial band (CK-MB) or troponin T. Electrocardiogram showed sinus tachycardia and low-voltage QRS complexes (**Figure 2A**), while transthoracic echocardiography (TTE) revealed pericardial effusion. We diagnosed her with cardiac tamponade, and she underwent pericardiocentesis, removing 500 ml of bloody fluid by drainage tube. Subsequently, her symptoms and tachycardia improved, and her oxygen saturation was 96% without oxygen administration. Cytology from the pericardial fluid revealed class V and neoplastic cells consistent with metastatic lung adenocarcinoma but no genetic mutation was detected by highly sensitive next-generation sequencing gene panel assay. The pericardial fluid drainage tube was removed 6 days later since there was no re-accumulation of pericardial fluid.

A second cycle of chemotherapy was administered 3 weeks after the first cycle since her blood tests revealed declining tumor markers, and no regrowth of the primary tumor on CT scan. Twenty-two days after admission, she developed a mild sore throat and tested positive for SARS-CoV-2 antigen. She was diagnosed with mild COVID-19, isolated, and treated with sotrovimab. Twenty-seven days after admission, blood tests showed elevated CK, CK-MB and troponin T, and a negative T wave appeared on electrocardiogram (**Figure 2B**). She did not complain of palpitations or chest pain. TTE showed a left ventricular ejection fraction of about 70%, with no re-accumulation of pericardial fluid, ventricular wall thickening, or ventricular hypokinesis. However, CK and CK-MB continued to rise on blood tests, and palpitations appeared 5 days later. She was afebrile with a blood pressure of 118/82 mm Hg, a heart rate of 111 beats/min, and an oxygen saturation of 93% on 3 liters per minute of oxygen administration. Differential diagnoses were considered, with myocarditis most concerning, followed by acute coronary syndrome, takotsubo cardiomyopathy, pericardial effusion, and pulmonary embolism. An electrocardiogram showed monomorphic VT (**Figure 2C**). Blood tests revealed CK 5906 U/l, CK-MB 141.7 ng/ml, troponin T 0.721 ng/ml, and N-terminal prohormone of brain natriuretic peptide (NT-proBNP) 1368 pg/ml. Pulmonary embolism was subsequently ruled out with computed tomography–angiography of the chest. Her hemodynamics had been stable, and she was started on continuous intravenous amiodarone. The next day, coronary angiography and endomyocardial biopsy (EMB) were performed. Her coronary arteries were found to be normal. Cardiac magnetic resonance (CMR) was not performed due to infection control.

**Figure 3:**
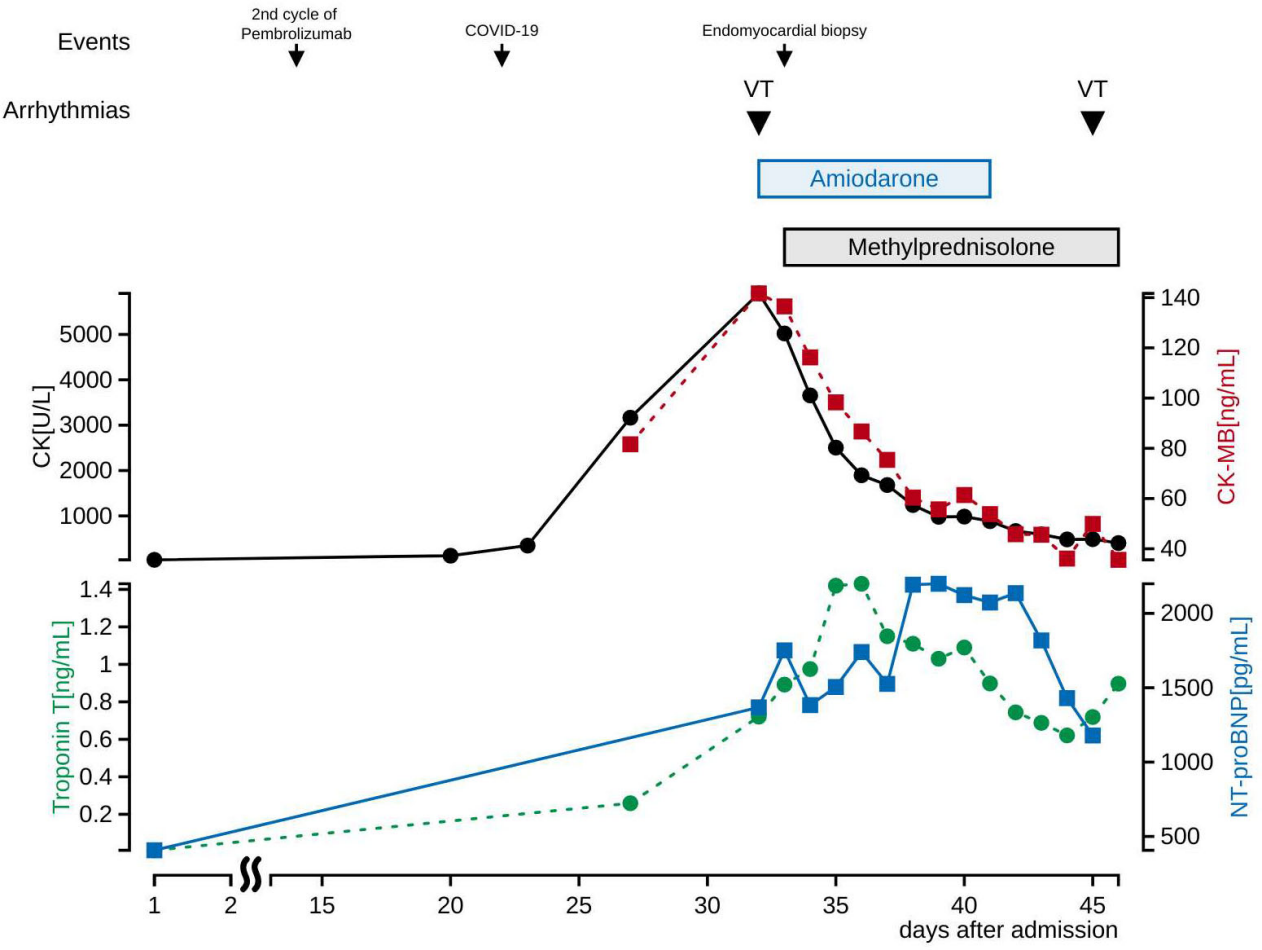
Cardiac biomarkers and electrolytes in blood tests after admission. CK and CK-MB decreased initially after methylprednisolone administration, and troponin T and NT-proBNP decreased later. Electrolytes values at the first occurrence of VT were K 4.1 mEq/L, Ca 9.1 mg/dL, Mg 1.8 mg/dL, and at the second occurrence of VT were K 4.7 mEq/L, Ca 8.9 mg/dL, Mg 2.0 mg/dL. Drugs that induce VT are not used.

**Figure 4:**
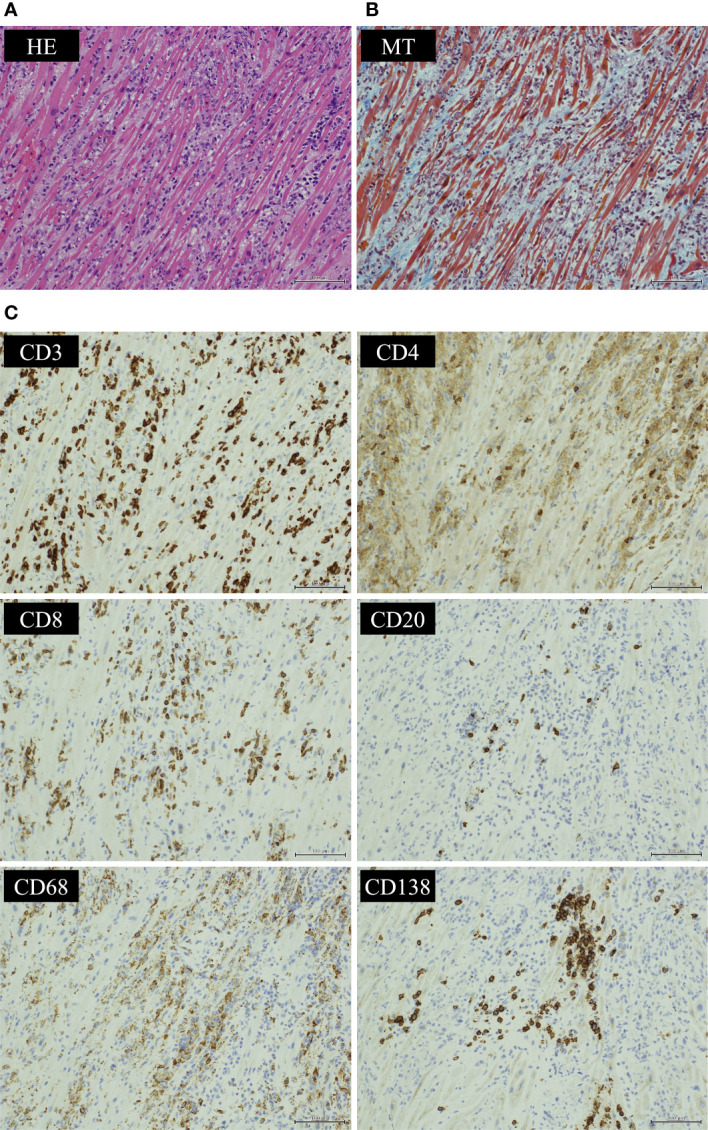
Histological findings of endocardial biopsy. Histological findings of the myocardium showed **(A)** an infiltrate of inflammatory cells predominantly composed of lymphocytes by hematoxylin-eosin (HE) staining and **(B)** granulation fibrosis of the stroma by Masson’s trichrome (MT) staining. Immunostaining of the tissue showed **(C)** CD3-positive T cells infiltrated more than CD20-positive B cells. CD8-positive T lymphocytes infiltrated more than CD4-positive T lymphocytes. Infiltration of CD68-positive macrophages and CD138-positive plasma cells was also observed. (HE ×200, MT ×200 and CD3/CD4/CD8/CD20/CD68/CD138 immunostaining ×200).

We suspected pembrolizumab-induced myocarditis and initiated daily methylprednisolone (1 mg/kg/day) immediately after EMB. The following day, CK and CK-MB decreased on blood test (**Figure 3**) and ventricular tachycardia disappeared on electrocardiogram. Eight days after the start of methylprednisolone administration, negative T waves remained on electrocardiogram, but ST-segment elevation was no longer present (**Figure 2D**); therefore, she was considered to have passed the acute stage and amiodarone administration was terminated. Nine days after EMB, she was diagnosed histologically as having acute lymphocytic myocarditis. Myocardial tissue collected at the EMB showed an infiltrate of inflammatory cells predominantly composed of lymphocytes (**Figure 4A**) and granulation fibrosis of the stroma (**Figure 4B**). Immunostaining of the tissue showed an inflammatory cell infiltrate predominantly composed of CD8-positive T lymphocytes (**Figure 4C**). A viral genome study of the tissue was not available at our institution. Twelve days after the start of methylprednisolone administration, R-on-T phenomenon triggered polymorphic VT (**Figure 2E**). It immediately degenerated into ventricular fibrillation and cardiopulmonary resuscitation was attempted, but she died.

## Discussion

This is the first report of pembrolizumab-induced myocarditis after COVID-19 infection and is also a valuable case in which electrocardiographic changes of myocarditis could be recorded in detail. Although the patient died as a result of arrhythmia, we were able to confirm that corticosteroids are markedly effective in the acute phase of pembrolizumab-induced myocarditis.

Myocarditis is an inflammatory disease of the myocardium caused by viral infection, autoimmunity, or drugs (8). The definitive diagnosis of myocarditis is made by EMB. Myocarditis is classified as eosinophilic, lymphocytic, giant cell, granulomatous or pleomorphic based on the type of cells infiltrating the myocardium. In recent years, ICI-induced myocarditis has been reported with the spread of ICI, and COVID-19-associated myocarditis with the COVID-19 pandemic.

ICI-induced myocarditis occurs when ICIs maintain T lymphocyte activity, T lymphocytes infiltrate the myocardium, and the immune response is excessive (9). The median time of onset was reported to be 34 days after the first ICI administration (3). Histological findings of EMB have been reported to show myocardial infiltration of CD4- positive lymphocytes, CD8-positive lymphocytes, and CD68-positive macrophages (10–12). Corticosteroids are often used in the initial treatment of ICI-induced myocarditis, and other immunosuppressive agents are also considered in corticosteroid-resistant patients (11, 12, 13). Guidelines published in 2018 by the American Society of Clinical Oncology and the National Comprehensive Cancer Center Network recommend treatment with 1 to 2 mg/kg of prednisone for ICI-induced myocarditis (15).

On the other hand, COVID-19-associated myocarditis is thought to result from direct damage to the myocardium by the virus and myocardial damage by the host’s immune response (16). The exact incidence of COVID-19-associated myocarditis is unknown because of diagnostic difficulties; some reports indicate that 5.0% of COVID-19 patients developed new onset myocarditis (17). Fulminant myocarditis caused by COVID-19 has been reported to produce ventricular dysfunction and heart failure within 2 to 3 weeks after infection with SARS-CoV-2 (18, 19). Histological findings of EMB shows infiltration of CD4- and CD8-positive lymphocytes in myocardial tissue, as well as CD68-positive macrophages in patients with severe clinical symptoms, such as fulminant myocarditis (20, 21). There are reports that the SARS-CoV-2 genome was detected in myocardial tissue from some COVID-19 patients (22–25). However, there have been reports of virus-negative COVID-19-associated myocarditis, and the authenticity of the SARS-CoV-2 genome remains uncertain (26). Although the treatment of COVID-19-associated myocarditis has not yet been established, corticosteroids are not recommended in viral myocarditis (27).

This patient developed myocarditis 41 days after the first dose of pembrolizumab and 11 days after SARS-CoV-2 infection. Myocardial tissue showed histological findings of acute lymphocytic myocarditis. The timing of onset and histological findings of myocarditis were consistent with both pembrolizumab-induced myocarditis and COVID-19-associated myocarditis. We considered pembrolizumab-induced myocarditis most likely since that myocarditis improved markedly after corticosteroid administration. However, COVID-19-associated myocarditis also causes myocardial damage due to the immune response, so it cannot be completely ruled out. COVID-19 infection has been reported to increase the risk of serious irAEs and may have triggered the development of pembrolizumab-induced myocarditis in this case (28).

An electrocardiogram is a simple test that records the heart’s electrical signals and is often used to detect arrhythmias and myocardial disorders. In this case, symptoms of myocarditis, such as palpitations and chest pain, were not present at first, and it was difficult to suspect myocarditis from the symptoms alone. However, we were able to suspect myocarditis at an early stage based on elevated CK and electrocardiographic changes. In addition, frequent ECG testing after the onset of myocarditis made it possible to document ECG changes during the course of treatment for myocarditis. Poor prognostic factors in electrocardiograms of acute myocarditis have been reported as pathological Q wave, wide QRS complex, QRS/T angle ≥ 100°, prolonged QT interval, high-degree atrioventricular block and malignant ventricular tachyarrhythmia (29–32). There are also reports of a high incidence of heart block, such as complete atrioventricular block and right bundle branch block, in electrocardiograms of patients with ICI-induced myocarditis (33). Her ECG showed no heart block, but a wide QRS complex, QRS/T angle ≥100°, prolonged QT interval and malignant ventricular tachyarrhythmia, which predicted a poor prognosis.

There have been several case reports of successful treatment of pembrolizumab-induced myocarditis (34–36). However, in this case, she survived the acute stage of myocarditis without the use of an extracorporeal circulatory device, but the resulting arrhythmia in the post-acute stage resulted in her death. It is suggested that the arrhythmia was caused by severe myocardial damage due to acute myocarditis. The reason for the severe myocardial damage may be related to COVID-19 infection and pericardial invasion of lung cancer. This patient had been infected with COVID-19 prior to the onset of myocarditis, so infection control measures were necessary. This limited the types of tests that could be performed and delayed the diagnosis of myocarditis. It also took longer to respond to emergencies, making it difficult to deal with fatal arrhythmias. In addition, the possibility of COVID-19-associated myocarditis was considered at the pre-treatment stage, which caused a delay in the initiation of corticosteroid administration. There is a report of pembrolizumab-induced myocarditis in a patient with pericardial infiltration of lung cancer (37). Thus, the administration of ICI to patients with pericardial infiltration of tumor may have resulted in excessive lymphocyte infiltration into the myocardium.

There are several limitations in the present case report. First, it was difficult to perform an CMR on COVID-19-infected patients at our institution, and second, we were unable to perform a viral genome study of myocardial tissue. A viral genome study of myocardial tissue might have brought us closer to identifying the cause of myocarditis.

## Conclusion

We report a case of pembrolizumab-induced myocarditis that developed after COVID-19 infection, in which we were able to document the course of electrocardiographic changes from onset to death. In myocarditis, elevated myocardial markers and electrocardiographic changes may precede clinical symptoms, so regular myocardial marker measurements and electrocardiographic testing are important. In addition, ECG examination is useful even after the start of treatment, since the prognosis may be inferred from ECG changes. Early diagnosis of pembrolizumab-induced myocarditis is important because early administration of corticosteroids may improve the prognosis. The impact of viral infections such as COVID-19 on patients with ICIs is unknown, and the appearance of irAEs after infection should be noted.

## Data availability statement

The original contributions presented in the study are included in the article. Further inquiries can be directed to the corresponding author.

## Author contributions

The manuscript was drafted by KN and KM. KN, KM, YS, JU, ST, HT, SA, AI, and MM examined and treated the patient. NY performed the histopathological assessment. YA gave clinical advice. All authors contributed to the article and approved the submitted version.

## References

[1] Das S Johnson DB. Immune-related adverse events and anti-tumor efficacy of immune checkpoint inhibitors. J Immunother Cancer (2019) 7(1):306. doi: 10.1186/s40425-019-0805-8 31730012 PMC6858629

[2] Wang DY Salem JE Cohen JV Chandra S Menzer C Ye F. Fatal toxic effects associated with immune checkpoint inhibitors: A systematic review and meta-analysis. JAMA Oncol (2018) 4(12):1721–8. doi: 10.1001/jamaoncol.2018.3923 PMC6440712 30242316

[3] Mahmood SS Fradley MG Cohen JV Nohria A Reynolds KL Heinzerling LM. Myocarditis in patients treated with immune checkpoint inhibitors. J Am Coll Cardiol (2018) 71(16):1755–64. doi: 10.1016/j.jacc.2018.02.037 PMC6196725 29567210

[4] Rodríguez-Abreu D Powell SF Hochmair MJ Gadgeel S Esteban E Felip E. Pemetrexed plus platinum with or without pembrolizumab in patients with previously untreated metastatic nonsquamous NSCLC: Protocol-specified final analysis from KEYNOTE-189. Ann Oncol (2021) 32(7):881–95. doi: 10.1016/j.annonc.2021.04.008 33894335

[5] Ammirati E Frigerio M Adler ED Basso C Birnie DH Brambatti M. Management of acute myocarditis and chronic inflammatory cardiomyopathy: An expert consensus document. Circ Heart Fail (2020) 13(11):e007405. doi: 10.1161/CIRCHEARTFAILURE.120.007405 33176455 PMC7673642

[6] Jiménez-Alejandre R Ruiz-Fernández I Martín P. Pathophysiology of immune checkpoint inhibitor-induced myocarditis. Cancers (Basel) (2022) 14(18):4494. doi: 10.3390/cancers14184494 36139654 PMC9497311

[7] Palaskas N Lopez-Mattei J Durand JB Iliescu C Deswal A. Immune checkpoint inhibitor myocarditis: Pathophysiological characteristics, diagnosis, and treatment. J Am Heart Assoc (2020) 9(2):e013757. doi: 10.1161/JAHA.119.013757 31960755 PMC7033840

[8] Matzen E Bartels LE Løgstrup B Horskær S Stilling C Donskov F. Immune checkpoint inhibitor-induced myocarditis in cancer patients: a case report and review of reported cases. Cardiooncology (2021) 7(1):27. doi: 10.1186/s40959-021-00114-x 34365980 PMC8351114

[9] Sobol I Chen CL Mahmood SS Borczuk AC. Histopathologic characterization of myocarditis associated with immune checkpoint inhibitor therapy. Arch Pathol Lab Med (2020) 144(11):1392–6. doi: 10.5858/arpa.2019-0447-OA PMC8445131 32150459

[10] De Luca G Campochiaro C Sartorelli S Peretto G Dagna L. Therapeutic strategies for virus-negative myocarditis: A comprehensive review. Eur J Intern Med (2020) 77:9–17. doi: 10.1016/j.ejim.2020.04.050 32402564

[11] Frustaci A Russo MA Chimenti C. Randomized study on the efficacy of immunosuppressive therapy in patients with virus-negative inflammatory cardiomyopathy: The TIMIC study. Eur Heart J (2009) 30(16):1995–2002. doi: 10.1093/eurheartj/ehp249 19556262

[12] Brahmer JR Lacchetti C Schneider BJ Atkins MB Brassil KJ Caterino JM. Management of immune-related adverse events in patients treated with immune checkpoint inhibitor therapy: American society of clinical oncology clinical practice guideline. J Clin Oncol (2018) 36(17):1714–68. doi: 10.1200/JCO.2017.77.6385 PMC6481621 29442540

[13] Agdamag ACC Edmiston JB Charpentier V Chowdhury M Fraser M Maharaj VR. Update on COVID-19 myocarditis. Medicina (Kaunas). (2020) 56(12):678. doi: 10.3390/medicina56120678 33317101 PMC7764165

[14] Buckley BJR Harrison SL Fazio-Eynullayeva E Underhill P Lane DA Lip GYH. Prevalence and clinical outcomes of myocarditis and pericarditis in 718,365 COVID-19 patients. Eur J Clin Invest. (2021) 51(11):e13679. doi: 10.1111/eci.13679 34516657 PMC8646627

[15] Kociol RD Cooper LT Fang JC Moslehi JJ Pang PS Sabe MA. Recognition and initial management of fulminant myocarditis: A scientific statement from the American heart association. Circulation (2020) 141(6):e69–92. doi: 10.1161/CIR.0000000000000745 31902242

[16] Siripanthong B Nazarian S Muser D Deo R Santangeli P Khanji MY. Recognizing COVID-19-related myocarditis: The possible pathophysiology and proposed guideline for diagnosis and management. Heart Rhythm (2020) 17(9):1463–71. doi: 10.1016/j.hrthm.2020.05.001 PMC7199677 32387246

[17] Jaiswal V Sarfraz Z Sarfraz A Mukherjee D Batra N Hitawala G. COVID-19 infection and myocarditis: A state-of-the-Art systematic review. J Prim Care Community Health (2021) 12:21501327211056800. doi: 10.1177/21501327211056800 34854348 PMC8647231

[18] Fox SE Li G Akmatbekov A Harbert JL Lameira FS Brown JQ. Unexpected features of cardiac pathology in COVID-19 infection. Circulation (2020) 142(11):1123–5. doi: 10.1161/CIRCULATIONAHA.120.049465 32689809

[19] Castiello T Georgiopoulos G Finocchiaro G Claudia M Gianatti A Delialis D. COVID-19 and myocarditis: A systematic review and overview of current challenges. Heart Fail Rev (2022) 27(1):251–61. doi: 10.1007/s10741-021-10087-9 PMC7988375 33761041

[20] Escher F Pietsch H Aleshcheva G Bock T Baumeier C Elsaesser A. Detection of viral SARS-CoV-2 genomes and histopathological changes in endomyocardial biopsies. ESC Heart Fail (2020) 7(5):2440–7. doi: 10.1002/ehf2.12805 PMC7307078 32529795

[21] Peretto G Villatore A Rizzo S Esposito A De Luca G Palmisano A. The spectrum of COVID-19-Associated myocarditis: A patient-tailored multidisciplinary approach. J Clin Med (2021) 10(9):1974. doi: 10.3390/jcm10091974 34064463 PMC8124580

[22] Bearse M Hung YP Krauson AJ Bonanno L Boyraz B Harris CK. Factors associated with myocardial SARS-CoV-2 infection, myocarditis, and cardiac inflammation in patients with COVID-19. Mod Pathol (2021) 34(7):1345–57. doi: 10.1038/s41379-021-00790-1 PMC9813560 33727695

[23] Del Nonno F Frustaci A Verardo R Chimenti C Nicastri E Antinori A. Virus-negative myopericarditis in human coronavirus infection: Report from an autopsy series. Circ Heart Fail (2020) 13(11):CIRCHEARTFAILURE120007636. doi: 10.1161/CIRCHEARTFAILURE.120.007636 33176456 PMC7673636

[24] Chen HS Wang W Wu SN Liu JP. Corticosteroids for viral myocarditis. Cochrane Database Syst Rev (2013) 2013(10):CD004471. doi: 10.1002/14651858.CD004471.pub3 24136037 PMC8094275

[25] Guo M Liu J Miao R Ahmed Z Yu J Guan J. A single center retrospective study of the impact of COVID-19 infection on immune-related adverse events in cancer patients receiving immune checkpoint inhibitors. J Immunother (2022) 45(9):389–95. doi: 10.1097/CJI.0000000000000440 PMC9528807 36066505

[26] Buttà C Zappia L Laterra G Roberto M. Diagnostic and prognostic role of electrocardiogram in acute myocarditis: A comprehensive review. Ann Noninvasive Electrocardiol (2020) 25(3):e12726. doi: 10.1111/anec.12726 31778001 PMC7958927

[27] Ammirati E Veronese G Brambatti M Merlo M Cipriani M Potena L. Fulminant versus acute nonfulminant myocarditis in patients with left ventricular systolic dysfunction. J Am Coll Cardiol (2019) 74(3):299–311. doi: 10.1016/j.jacc.2019.04.063 31319912

[28] Hung Y Lin WH Lin CS Cheng SM Tsai TN Yang SP. The prognostic role of QTc interval in acute myocarditis. Acta Cardiol Sin (2016) 32(2):223–30. doi: 10.6515/acs20150226a PMC4816921 27122953

[29] Kindermann I Barth C Mahfoud F Ukena C Lenski M Yilmaz A. Update on myocarditis. J Am Coll Cardiol (2012) 59(9):779–92. doi: 10.1016/j.jacc.2011.09.074 22361396

[30] Power JR Alexandre J Choudhary A Ozbay B Hayek S Asnani A. Electrocardiographic manifestations of immune checkpoint inhibitor myocarditis. Circulation (2021) 144(18):1521–3. doi: 10.1161/CIRCULATIONAHA.121.055816 PMC8567307 34723640

[31] Su L Liu C Wu W Cui Y Wu M Chen H. Successful therapy for myocarditis concomitant with complete heart block after pembrolizumab treatment for head and neck squamous cell carcinoma: A case report with literature review. Front Cardiovasc Med (2022) 9:898756. doi: 10.3389/fcvm.2022.898756 35647073 PMC9133913

[32] Schiopu SRI Käsmann L Schönermarck U Fischereder M Grabmaier U Manapov F. Pembrolizumab-induced myocarditis in a patient with malignant mesothelioma: plasma exchange as a successful emerging therapy-case report. Transl Lung Cancer Res (2021) 10(2):1039–46. doi: 10.21037/tlcr-20-1095 PMC7947381 33718042

[33] Wang Q Hu B. Successful therapy for autoimmune myocarditis with pembrolizumab treatment for nasopharyngeal carcinoma. Ann Transl Med (2019) 7(11):247. doi: 10.21037/atm.2019.04.73 31317017 PMC6603355

[34] Bersanelli M. Controversies about COVID-19 and anticancer treatment with immune checkpoint inhibitors. Immunotherapy (2020) 12(5):269–73. doi: 10.2217/imt-2020-0067 PMC7117596 32212881

[35] Yongzhi X. COVID-19-associated cytokine storm syndrome and diagnostic principles: an old and new issue. Emerg Microbes Infect (2021) 10(1):266–76. doi: 10.1080/22221751.2021.1884503 PMC7894425 33522893

[36] Pasrija R Naime M. The deregulated immune reaction and cytokines release storm (CRS) in COVID-19 disease. Int Immunopharmacol (2021) 90:107225. doi: 10.1016/j.intimp.2020.107225 33302033 PMC7691139

[37] Takashi O Akira S Yoshiki S Tsuyoshi T Shunzo H. Fulminant myocarditis after initial pembrolizumab treatment for squamous cell carcinoma of the lung. an autopsy case report. Japanese J Lung Cancer (2020) 60:335–40. doi: 10.2482/haigan.60.335

